# A comparative study of mesenchymal stem cell transplantation and NTG-101 molecular therapy to treat degenerative disc disease

**DOI:** 10.1038/s41598-021-94173-w

**Published:** 2021-07-20

**Authors:** Ajay Matta, Muhammad Zia Karim, Hoda Gerami, Bettina Benigno, W. Mark Erwin

**Affiliations:** 1 Notogen Inc., Toronto, Ontario Canada; 2 Department of Surgery, University of Toronto, Toronto, Ontario Canada; 3 Canadian Memorial Chiropractic College, Toronto, Ontario Canada

**Keywords:** Stem cells, Mesenchymal stem cells

## Abstract

Cellular replacement therapy using mesenchymal stem cells (MSCs) and/or the delivery of growth factors are at the forefront of minimally invasive biological treatment options for Degenerative Disc Disease (DDD). In this study, we compared the therapeutic potential of a novel drug candidate, NTG-101 to MSCs, including rat cartilage derived stem cells (rCDSCs), bone marrow stem cells (rBMSCs) and human Umbilical Cord Derived Mesenchymal Stem Cells (hUCMSCs) for the treatment of DDD. We induced DDD using a validated image-guided needle puncture injury in rat-tail IVDs. Ten weeks post-injury, animals were randomized and injected with MSCs, NTG-101 or vehicle. At the end of the study, histological analysis of the IVD-Nucleus Pulposus (NPs) injected with NTG-101 or rCDSCs showed a healthy or mild degenerative phenotype in comparison to vehicle controls. Immunohistochemical analysis revealed strong expression of aggrecan, collagen 2, brachyury and Oct4 in IVD-NPs injected with NTG-101. Our results also demonstrated suppression of inflammation induced p38 and NFκB resulting in inhibition of catabolic genes, but activation of Smad-2/3, Erk-1/2 and Akt-dependent signaling inducing anabolic genes in IVD-NP on treatment with NTG-101. In conclusion, a single injection of NTG-101 into the degenerative disc demonstrated superior benefits compared to stem cell transplantation.

## Introduction

Degenerative disc disease (DDD) is often associated with chronic back pain in humans and involves loss of viable cells, and degradation of the healthy extracellular matrix (ECM) that results from an imbalance in anabolic and catabolic factors within intervertebral disc-nucleus pulposus (IVD-NP)^1–4^. Aging, trauma, excessive mechanical loading, and genetic factors influence disc degeneration involving cellular senescence and death, impaired proteoglycan expression, and the increased expression of matrix degrading enzymes and pro-inflammatory cytokines^1, 3, 5, 6^. It has been reported that IVD-NP cells are capable of secreting pro-inflammatory cytokines including Interleukin-1 beta (IL-1β) and Tumor Necrosis Factor alpha (TNF-α) under biomechanical stress^7, 8^. Interleukin-1 beta (IL-1β) has been reported to be a master regulator in the degenerative process that induces the expression of matrix degrading enzymes (MMP-3, MMP-9, MMP-13), aggrecanases (ADAMTS-4/5) and expression of other pro-inflammatory cytokines (IL-6, IL-8, TNF-α) both in vitro and in vivo^9–13^. In a healthy IVD, the catabolic activity of IL-1β is regulated by the presence of its natural inhibitor, Interleukin-1 receptor antibody (IL-1Ra). But the onset and progression of DDD is a result of over-expression of IL-1β, tipping the balance towards a catabolic, pro-inflammatory harsh IVD-NP microenvironment that is implicated in discogenic pain^8–15^. At present, DDD associated lower back pain is treated conservatively with analgesics, non-steroid anti-inflammatory drugs, physical therapies or in some cases by surgical intervention^16–18^.

In humans, a healthy IVD-NP is rich in proteoglycans such as aggrecan as well as collagen type 2 that resist loading forces placed upon the spine. Changes in lifestyle, aging and gene expression lead to chronic inflammation resulting in the loss of NP cells, triggering the development and progression of DDD^19, 20^. In humans, the IVD-NP is rich in notochordal cells (NCs) and Nucleus Pulposus Progenitor Cells (NPPCs) from birth until early adolescence and are gradually replaced by chondrocyte like cells (CLCs). Sakai et al.^21^, demonstrated that IVD-specific progenitors are exhausted with ageing and degeneration of the IVD. The loss of IVD stem cells with ageing has been associated with a decrease in the ability for self-repair and regenerative potential. Thus, the loss of NP cells in conjunction with chronic inflammation and the development of a progressively harsh microenvironment (such as hypoxia, a reduced pH, and limited nutrient supply) poses a significant challenge for any biologically based therapy to treat DDD^22–25^. Nevertheless, cellular replacement or growth factor-based therapies capable of reducing inflammation, enabling cellular replacement and/or activating endogenous stem cells provides an attractive strategy for enhancing repair and regeneration of the degenerative disc^24–26^. Recently, using in vivo rat-tail and chondrodystrophic canine (CD canine) models of DDD we identified and demonstrated the therapeutic potential of a novel molecular therapeutic “NTG-101” containing a combination of recombinant human (rh) Connective Tissue Growth Factor (CTGF) and Transforming Growth Factor beta 1 (TGF-β1) within an excipient solution. A single intradiscal injection of NTG-101 restored the IVD-NP post-injury and maintained biomechanical properties in CD-canine IVDs, 14 weeks post-injection. Further, CD-canine degenerative IVDs injected with NTG-101 demonstrated no progression of DDD after needle puncture injury on MRI and radiographic analysis as compared to the vehicle injected IVDs at the endpoint that showed extensive degenerative changes^27^. Our study also demonstrated that NTG-101 induced recovery of the needle puncture-injured rat-tail IVD-NP by showing a similar expression of notochordal and stem cell rich NPs with robust aggrecan and Collagen type 2A1(Col2A1) within the ECM, similar to age-matched uninjured IVDs. Finally, NTG-101 injected IVDs showed reduced expression of inflammation and pain associated proteins (IL-1β, IL-6, IL-8, TNF-α, Cox-2 and MMP-13) clearly demonstrating the pro-anabolic and anti-inflammatory role of NTG-101 in degenerative IVD-NPs^27, 28^.

Mesenchymal stem cells (MSCs) with self-renewal capability and multi-lineage differentiation potential are the subject of many tissue engineering strategies with which to treat DDD. Mesenchymal stem cells (MSCs) derived from bone marrow (BMSCs), umbilical cord (UCMSCs), adipose tissues (ADPCs), and the IVD-NP (NP-MSCs) are among the candidate stem cells currently being evaluated in pre-clinical animal models and/or clinical trials (Phase 1/2/3) in DDD patients^29–34^. Several studies have reported restoration of disc height, T2-weighted signal intensity on MRI, improved histology, ECM gene expression and pain relief following transplantation of MSCs in pre-clinical models and human clinical trials^33–38^. With respect to the mechanism of action of stem cells, it has been postulated that BMSCs may differentiate into an NP cell-like phenotype, induce growth, or prevent cell death of endogenous NP cells via direct cell–cell contact or paracrine signaling^39, 40^. However, there are several challenges for the translation of stem cell-based therapeutics into the clinic^41, 42^. The harsh IVD micro-environment negatively influences the viability and metabolic activity of stem cells^41–44^. Furthermore, isolation of MSCs from adult sources requires invasive procedures, and these cells may have limited proliferation and differentiation potential due to aging. Moreover, the utility of autologous NP cells vs. allogeneic sources of MSCs for grafting and their efficacy for the regeneration of a degenerative IVD is limited, leaving the therapeutic potential of MSCs derived from different sources to induce a regenerative effect in the degenerative IVD yet to be determined.

In this study, we compared the therapeutic, NTG-101, with MSCs derived from three different sources, i.e., rat articular cartilage (rCDSCs), rat bone marrow (rBMSCs) and human umbilical cord (hUCMSCs), in vivo using a rat-tail disc needle puncture model of DDD. Our results showed that needle puncture injured IVDs subsequently injected with NTG-101 demonstrated superior characteristics as compared to an injection of MSCs (rCDSCs/rBMSCs/hUCMSCs) in the maintenance of a healthy phenotype. A single injection of NTG-101 yielded IVD-NP morphology, cellularity, and molecular phenotype like age-matched healthy specimens. However, intra-discal injections of MSCs showed limited potential to regenerate degenerative IVDs in comparison to NTG-101 for the treatment of DDD.

## Results

### Gene expression analysis of rat and human primary MSC stem cell cultures

**Figure 1:**
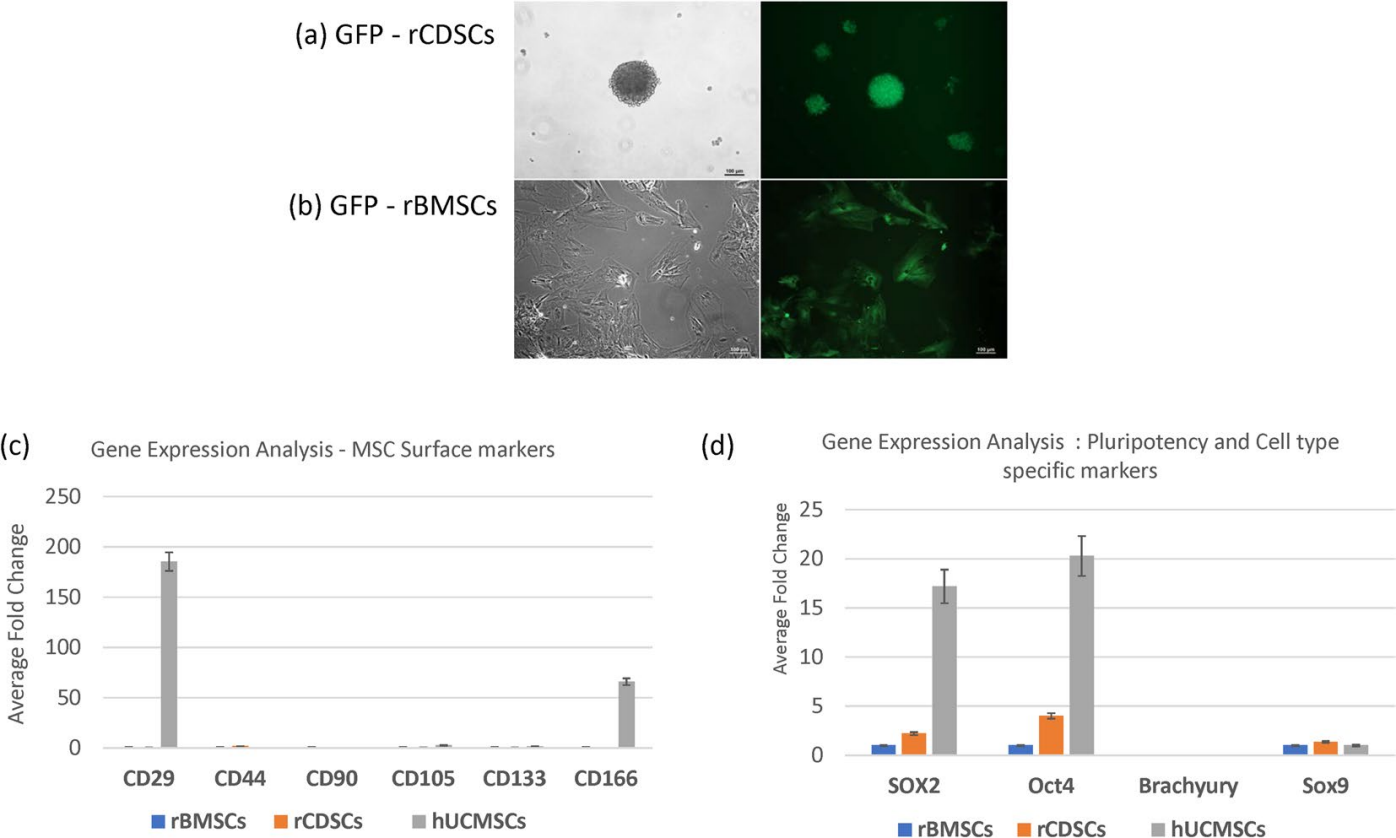
Panel represents a photomicrograph of (**a**) rCDSCs spheroids; (**b**) rBMSCs in adherent flasks in cell culture under bright field and green fluorescence setting using Nikon microscope (Nikon Eclipse TE2000-U, Scale bar: 100 µm). Total RNA was isolated from rCDSCs, rBMSCs and hUCMSCs and gene expression was determined using qPCR analysis. Histograms show expression level of (**c**) MSC surface markers (CD29, CD44, CD90, CD105, CD133 and CD166); (**d**) Stem cell markers (Oct4, Sox2), notochordal cell marker, Brachyury and chondrogenesis marker, Sox9 in rCDSCs and hUCMSCs with respect to rBMSCs used as a reference control.

Total RNA was extracted from MSCs (rCDSCs, rBMSCs, hUCMSCs) and the expression of MSC surface markers (CD29, CD44, CD90, CD105, CD133, CD166), pluripotency markers (Oct4, Sox2), chondrogenic marker (Sox9), notochordal cell marker (brachyury) and the hematopoietic progenitor cell markers (CD34, CD45) were determined using qPCR (Fig. 1a–d, Supplementary Tables S1 and S2). Our data showed the presence of MSC surface markers (CD29, CD44, CD90, CD105 and CD133) depicting mesenchymal cell characteristics of rCDSCs, rBMSCs and hUCMSCs (Fig. 1c, Supplementary Table S2). None of the MSCs (rCDSCs, rBMSCs and hUCMSCs) in cell culture showed detectable expression of the notochordal cell marker, brachyury, but expression of chondrocyte transcription factor, Sox9 was observed in all MSCs (Fig. 1d, Supplementary Table S1). As shown in Fig. 1c,d, hUCMSCs showed significantly higher expression of CD34, CD45, CD29, CD166, Sox2 and Oct4 genes in comparison to rBMSCs. No significant difference was observed in gene expression of MSC markers on rCDSCs as compared to rBMSCs (Fig. 1c). Low or no detectable expression of CD34 and CD45 was observed in rCDSCs, rBMSCs and hUCMSCs in cell culture demonstrating their lack of ability to differentiate into hematopoietic lineage (Supplementary Table S2).

### Immunofluorescence and Differentiation of Rat MSCs (rCDSCs/rBMSCs) in osteogenic, chondrogenic and adipogenic phenotype

**Figure 2:**
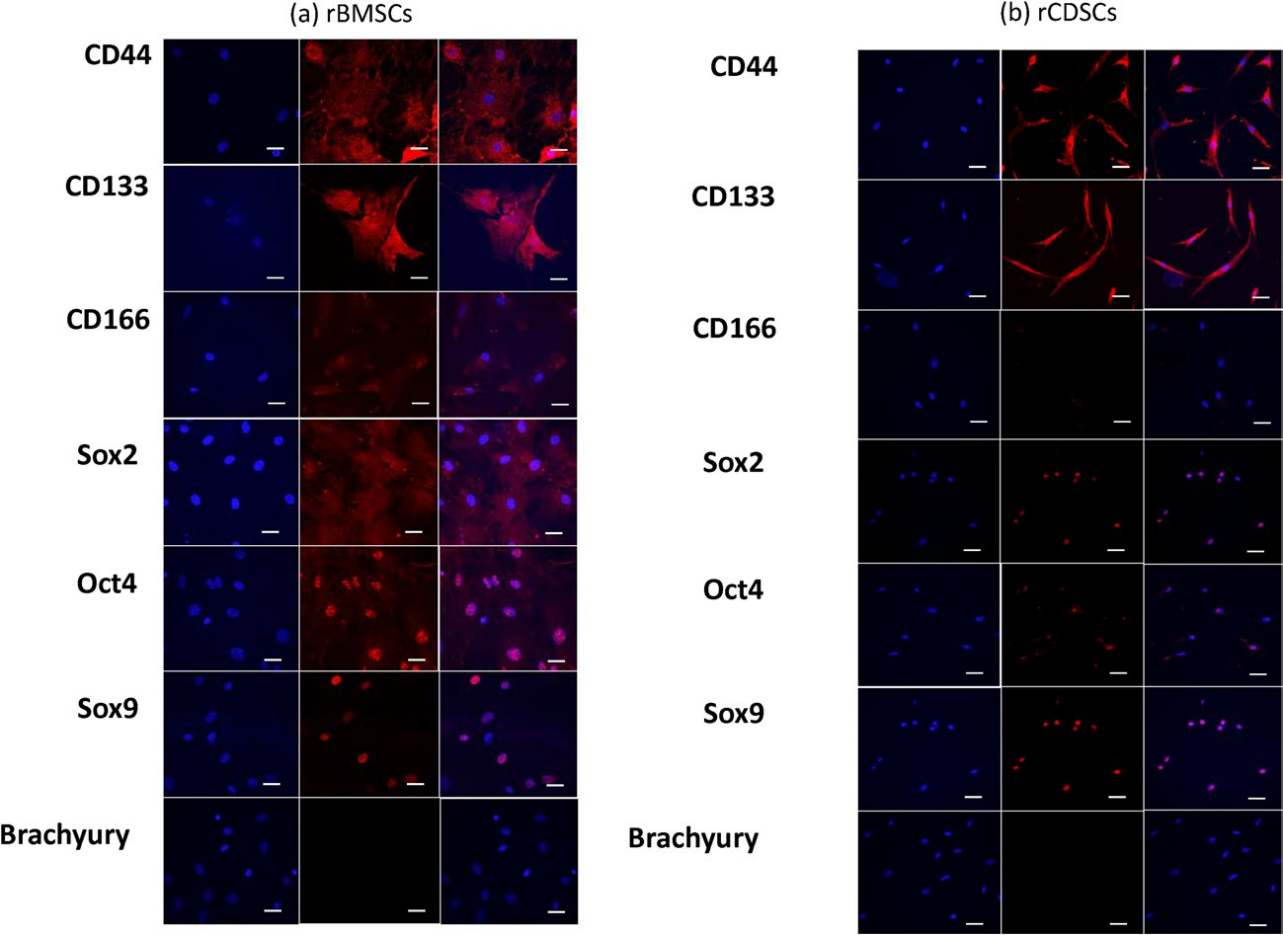
Confocal Laser Scan Microscopy (CLSM) of MSC surface markers and cell type specific protein markers in rat CDSCs and BMSCs. Rat cartilage and bone marrow derived mesenchymal stem cells were plated on matrigel coated glass coverslips and MSC surface markers (CD44, CD133 and CD166) and cell type specific protein markers (Sox2, Oct4, Sox9 and Brachyury) were analyzed using specific antibodies. The protein expression was detected using Alexa 568 fluorophore and DAPI was used as nuclear stain. As shown, panel shows cytoplasmic and membranous staining of CD44 and CD133 in (**a**) BMSCs and (**b**) CDSCs; CD166 expression was not observed in CDSCs, but membranous and diffused, faint cytoplasmic staining was observed BMSCs as shown in panel (**a**) and (**b**). As shown in **(a**) rBMSCs showed diffused cytoplasmic Sox2 staining, nuclear Oct4 and Sox9 (**b**) rCDSCs demonstrated nuclear staining of both pluripotency markers (Sox2, Oct4) and chondrocyte transcription factor (Sox9). Notochordal cell marker, Brachyury was not detected in both rBMSCs and rCDSCs as shown in the panel (Scale bar: 10 µm).

We determined protein expression and localization of MSC surface markers (CD44, CD133, CD166), plus nuclear transcription factors indicative of pluripotency (Oct4, Sox2), the chondrocyte transcription factor (Sox9) and notochordal cell marker (brachyury) in rCDSCs and rBMSCs. Immunofluorescence staining demonstrated faint, diffused cytoplasmic and/or distinct membranous staining of CD44, CD133 and CD166 in rat BMSCs, while rCDSCs showed staining for CD44 and CD133 only (Fig. 2a,b). Rat CDSCs showed nuclear expression of pluripotency markers, Oct4 and Sox2, whereas nuclear Oct4 and cytoplasmic Sox2 was observed in rBMSCs (Fig. 2). Both rCDSCs and rBMSCs showed nuclear expression of the chondrogenic transcription factor Sox9 with no detectable expression of brachyury (Fig. 2a,b).

To confirm the stemness characteristics of the cartilage and bone marrow derived rat MSCs, we differentiated these MSCs into osteogenic, chondrogenic and adipogenic lineages as defined in “Materials and methods”. Both rCDSCs and rBMSCs differentiated into osteogenic, chondrogenic and adipogenic lineages as shown by Alizarin red, Safranin O and Oil red O staining respectively confirming their stemness (Supplementary Fig. S1a,b).

### Histological evaluation of rat-tail IVD-NP tissue sections (healthy/PBS/NTG-101/MSCs)

**Figure 3:**
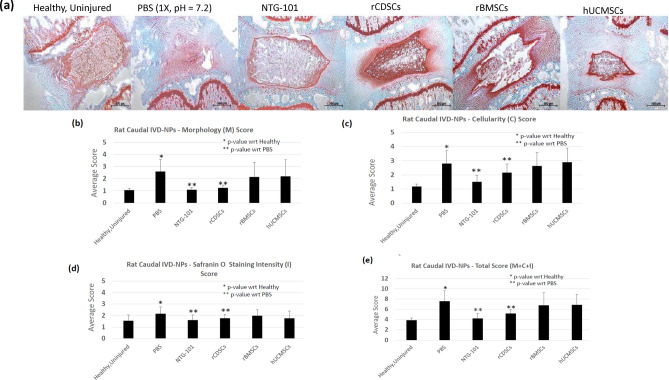
Histological analysis of rat caudal IVD-NPs. (**a**) Safranin O stained IVD-tissue sections derived from age-matched, uninjured IVDs and injured IVDs that received an intra-discal injection of PBS (1X, pH = 7.2), NTG-101, rCDSCs, rBMSCs or hUCMSCs. Histological images were acquired using bright field Nikon microscope (Nikon Eclipse TE2000-U, Scale bar: 500 µm). Panel shows the histograms representing average score of (**b**) Morphology (M), (**c**) Cellularity (C), (**d**) Safranin O staining intensity (I) and (**e**) average total score (M + C + I) for healthy, and injured IVDs injected with PBS (1×), NTG-101, rCDSCs, rBMSCs or hUCMSCs, 10 weeks post-injection (*,**p ≤ 0.05).

We injected the injured rat caudal IVD-NPs with vehicle (PBS), NTG-101, or MSCs (rCDSCs, rBMSCs and hUCMSCs) under fluoroscopic image guidance and after humane euthanasia, harvested the IVDs 10-weeks post-injection. We evaluated age matched healthy, uninjured, and treated discs tissue sections for histological features and scored them based upon morphology (M), cellularity (C) and Safranin-O staining intensity (I) (Table 1, Fig. 3a–e). Histological analysis showed a significant loss of cellularity in injured IVDs that received PBS (1×) demonstrating a degenerative phenotype (average total score, MCI = 7.6 ± 2.0, p < 0.001, Table 1, Fig. 3a–e). Rat IVDs injected with NTG-101 demonstrated mild/moderate Safranin O staining intensity (average total score, MCI = 4.2 ± 1.0) resembling age matched healthy IVDs (average total score, MCI = 3.9 ± 0.5, p < 0.001, Table 1, Fig. 3a–e). Safranin O staining of tissue sections obtained from rCDSC-injected rat-tail IVDs showed morphological and cellular intensity representing mild to moderate degeneration with an average total score, MCI = 5.2 ± 0.8 (p < 0.001, Table 1, Fig. 3a–e. In contrast, IVDs injected with rBMSCs showed moderate to severe degeneration i.e., fibrocartilaginous matrix with a loss of cells within the IVD-NP with an average total score, (MCI) of 6.8 ± 2.5 (p = 0.315, Table 1, Fig. 3a–e). Similarly, histological assessment of rat tail IVDs injected with hUCMSCs showed moderate to severe degeneration in IVDs with low cellularity and a fibrocartilaginous matrix (average total score, MCI = 6.9 ± 2.0, p = 0.315), similar to vehicle control (Table 1, Fig. 3a–e).

**Table 1:** Histological analysis of rat tail IVD-NPs.

	Morphology (M)	Cellularity (C)	Saf O intensity (I)	Total score = M + C + I
Average score (IVD-NP, n = 15)				
Healthy, uninjured	1.1 ± 0.1	1.2 ± 0.2	1.6 ± 0.5	3.9 ± 0.5
PBS	2.6 ± 1.0	2.8 ± 0.9	2.2 ± 0.6	7.6 ± 2.0
NTG-101	1.1 ± 0.2	1.5 ± 0.5	1.6 ± 0.5	4.2 ± 1.0
rCDSCs	1.2 ± 0.2	2.2 ± 0.6	1.8 ± 0.3	5.2 ± 0.8
rBMSCs	2.1 ± 1.2	2.6 ± 1.0	2.0 ± 0.5	6.8 ± 2.5
hUCMSCs	2.2 ± 1.4	2.9 ± 1.0	1.8 ± 0.6	6.9 ± 2.0
p-value				
PBS vs. Healthy	< 0.001	< 0.001	0.005	< 0.001
NTG-101 vs. PBS	< 0.001	< 0.001	0.007	< 0.001
rCDSCs vs. PBS	< 0.001	0.036	0.036	< 0.001
rBMSCs vs. PBS	0.267	0.635	0.387	0.315
hUCMSCs vs. PBS	0.365	0.778	0.081	0.315

### Immunohistochemical analysis of rat IVD-NP tissue sections (healthy/PBS/NTG-101/rCDSCs/rBMSCs/hUCMSCs)

**Figure 4:**
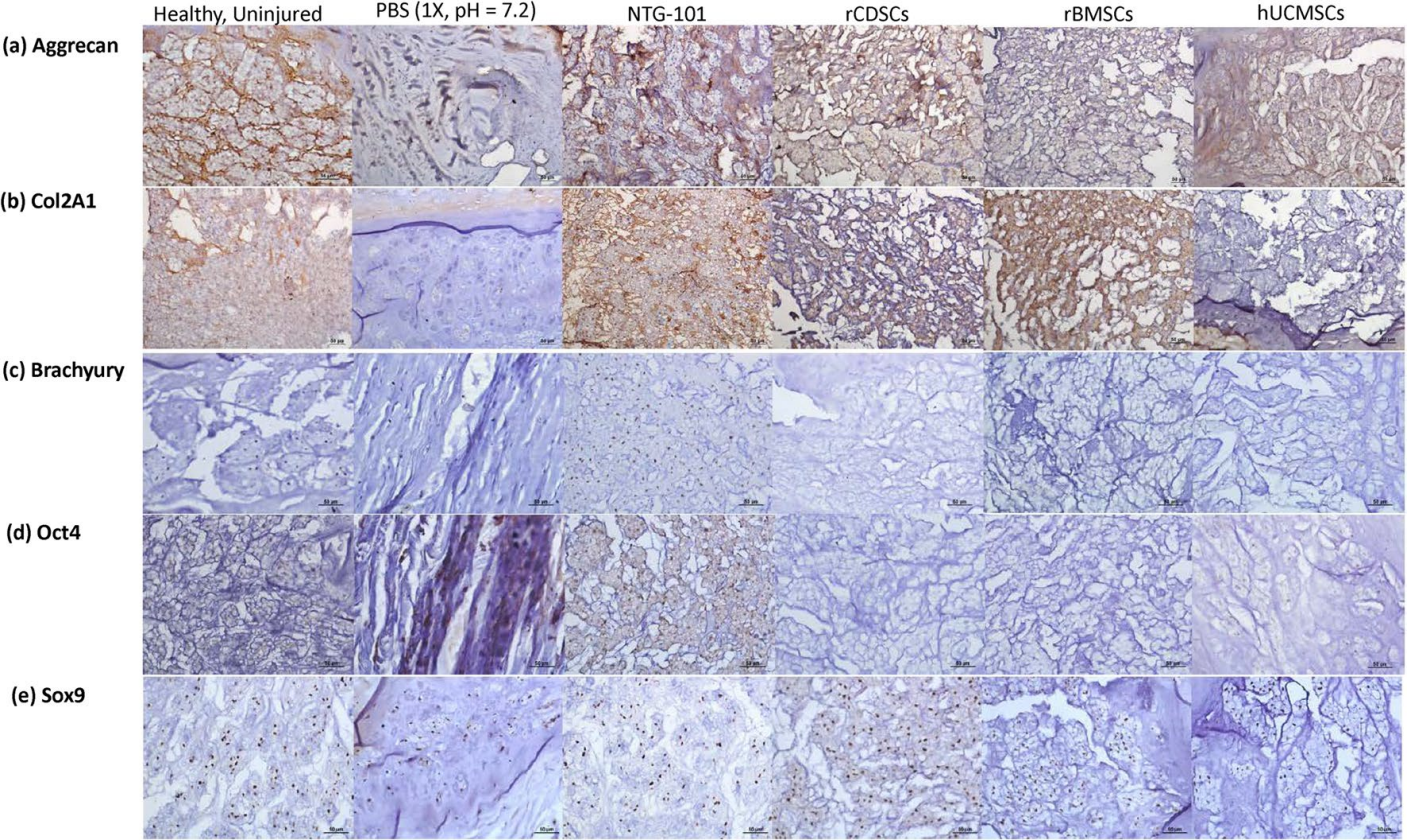
Immunohistochemical analysis of cell specific markers in in vivo rat tail needle puncture model of DDD. Panels show expression of healthy ECM proteins, (**a**) Aggrecan and (**b**) Col2A1 in age-matched, uninjured IVD-NPs, NTG-101, rCDSCs, rBMSCs or hUCMSCs injected IVD-NPs. Faint or loss of Aggrecan and Col2A1 expression was observed in PBS or hUCMSCs injected rat IVD-NPs; (**c**) nuclear expression of notochordal cell marker, Brachyury in age-matched, uninjured IVD-NPs and NTG-101 injected IVD-NPs. (**d**) Nuclear expression of stem cell marker, Oct4 in age-matched, uninjured IVD-NPs and NTG-101 injected IVD-NPs. Nuclear Brachyury or Oct4 was not observed in majority of the IVD-NPs injected with PBS or MSCs (rCDSC, rBMSCs or hUCMSCs); (**e**) Nuclear Sox9 was observed in uninjured, healthy IVD-NPs and IVDs injected with vehicle, NTG-101 or MSCs (rCDSC, rBMSCs or hUCMSCs. All images were acquired using bright field Nikon microscope (Nikon Eclipse TE2000-U, Scale bar: 50 µm).

**Figure 5:**
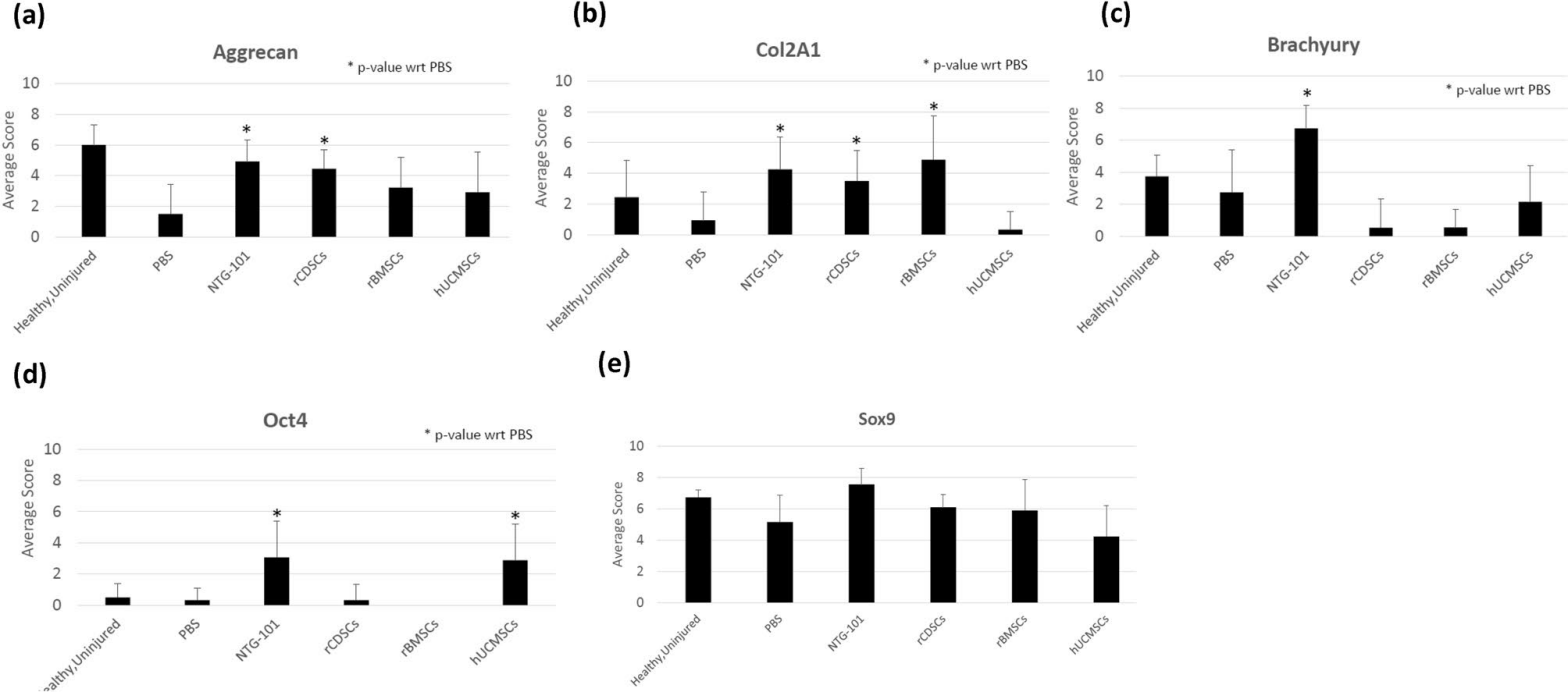
Panel shows the histograms representing average total score of immunohistochemical analysis of (**a**) Aggrecan, (**b**) Collagen 2A1, (**c**) Brachyury, (**d**) Oct4 and (**e**) Sox9 in rat IVD-NP tissue sections of healthy, and injured IVDs injected with PBS, NTG-101, rCDSCs, rBMSCs or hUCMSCs (*p ≤ 0.05).

Rat-tail IVDs injected with NTG-101 (Figs. 4a–d, 5a–d, Supplementary Fig. S3) showed moderate to intense expression of aggrecan and Col2A1 in the ECM, nuclear Brachyury and Oct4 representing a healthy, notochordal and stem cell rich IVD-NP. In contrast, rat tail IVDs injected with PBS (1×) developed a degenerative, fibrocartilaginous NP post-injury, with mild or no detectable expression of these proteins (Figs. 4a–d, 5a–d, Supplementary Fig. S3). Rat caudal IVDs that received an intra-discal injection of MSCs (rCDSCs/rBMSCs/hUCMSCs) showed differences in regenerative capabilities of MSCs derived from different sources. Rat caudal IVDs injected with rCDSCs showed moderate expression of aggrecan and Col2A1 but low or no detectable expression of Brachyury and Oct4 (Figs. 4a–d, 5a–d). However, rat-tail IVDs injected with rBMSCs showed low or no detectable expression of aggrecan, brachyury and Oct4 (Figs. 4a–d, 5a–d). Rat caudal IVDs injected with hUCMSCs 10 weeks post-injury, showed low or no detectable expression of aggrecan, Col2A1, and Brachyury (Figs. 4a–d, 5a–d), suggesting a limited regenerative capability. Further, we observed increased expression of Col2A1 within the ECM of IVD-NPs injected with rBMSCs and nuclear Oct4 immunostaining in only a few IVD-NP tissues injected with hUCMSCs (Figs. 4a–d, 5a–d). All IVD-NP tissue sections showed distinct, nuclear staining for the chondrogenic transcription factor, Sox9 in healthy and treated IVDs (Figs. 4e, 5e, Supplementary Fig. S3).

As described in “Materials and methods”, we used GFP-expressing rat MSCs (rCDSCs/rBMSCs) to track and detect these cells in rat tail IVDs post-injection. Our immunohistochemical analysis using GFP-specific mouse monoclonal antibodies demonstrated no detectable expression of GFP-/GFP-expressing cells in IVD-NPs injected with rCDSCs or rBMSCs 10 weeks post-injury (Supplementary Fig. S2). Green fluorescent protein (GFP) expressing Wistar rat tissue sections used as positive control (spine/caudal IVDs) showed strong membranous staining of GFP on NP-cells (Supplementary Fig. S2).

### Mechanism of action of NTG-101

**Figure 6:**
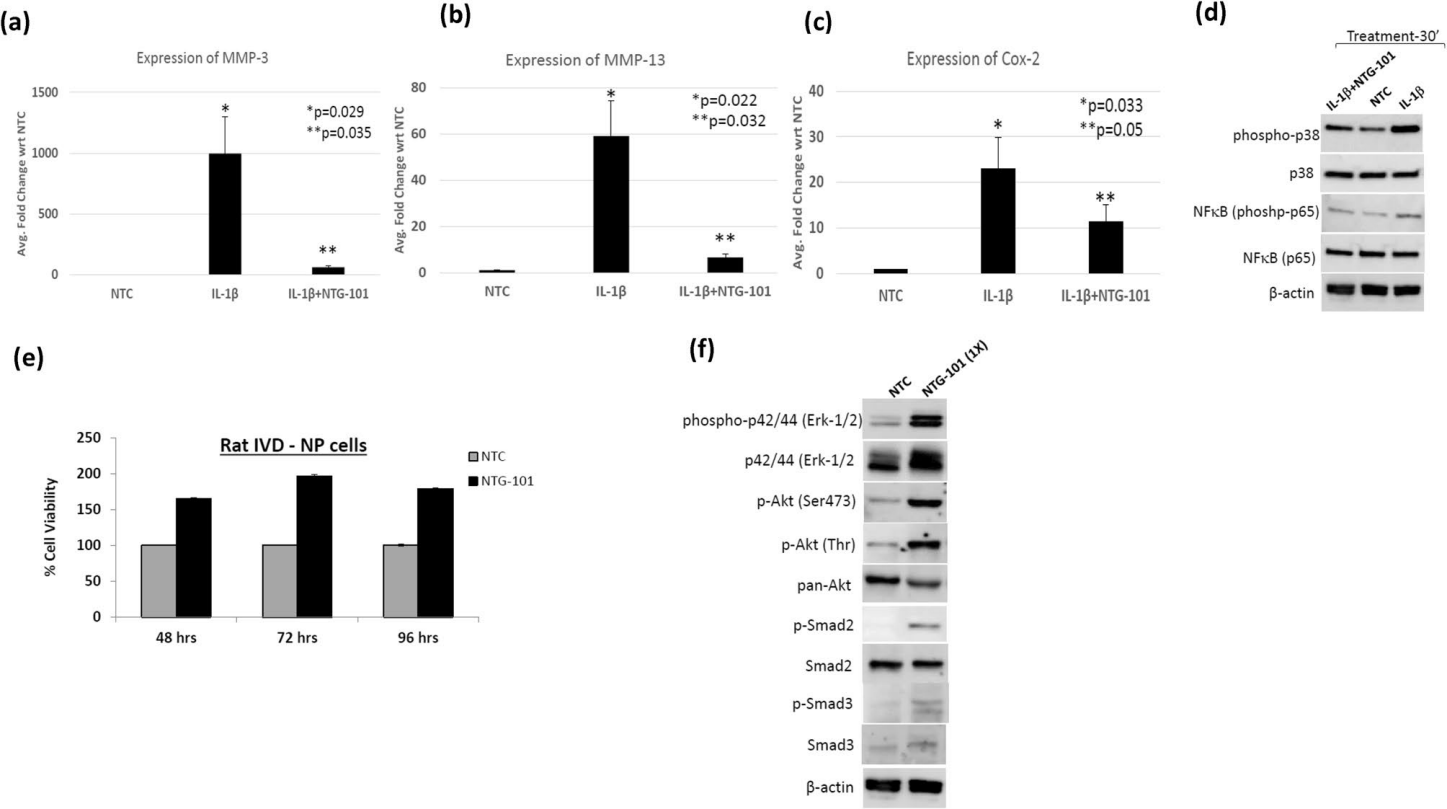
Panel shows histograms representing gene expression of (**a**) MMP-3, (**b**) MMP-13 and (**c**) Cox-2 in no treatment controls (NTC), rats NP cells treated with pro-inflammatory cytokine, IL-1β (10 ng/ml) alone or in the presence of NTG-101 for 24 h as determined using qPCR analysis (*p-value for IL-1β vs. NTC, **p p-value for (IL-1β + NTG-101) vs. IL-1β); (**d**) Western blot showing phosphorylation of p38 and p65 sub-unit of NFκB complex, total protein expression of p38, p65-NFκB and β-actin as loading control in cell lysates obtained from no treatment controls (NTC), cells treated with IL-1β (10 ng/ml) alone or in the in the presence of NTG-101 for 24 h; (**e**) Cell viability in rat IVD-NP cells treated with NTG-101 for 48–96 h with respect to no treatment controls (NTC); (**f**) Western blots showing increase in phosphorylation of p42/44, also known as Erk-1/2 (Thr202/Tyr204), Akt (Ser473), Akt (Thr308), Smad-2 (Ser465/467) and Smad-3 (Ser423/425) in whole cell lysates prepared from rat IVD-NP cells treated with NTG-101 for 30 min as compared to untreated, no treatment control cell lysates. Western blots of non-phosphorylated Erk-1/2, pan-Akt, Smad-2 and Smad-3 total protein served as a control for change in expression of protein in treated and control cells. β-actin served as loading control for Western blotting experiments.

Rat IVD-NP cells were treated with NTG-101 for varying intervals of time to elucidate the mechanism of action of NTG-101 in vitro. Inflammation is considered as the most important player in the development of a degenerative IVD. Treatment with the pro-inflammatory cytokine, IL-1β (10 ng/ml) for 24 h induced significant expression of ECM degrading enzymes (MMP-3, MMP-13) and the pain associated enzyme, cyclooxygenase-2 (Cox-2) in rat IVD-NP cells as compared to no treatment control (NTC) cells (Fig. 6a–c). However, the presence of NTG-101 significantly suppressed IL-1β induced expression of MMP-3, MMP-13 and Cox-2 within 24 h of treatment as revealed by qPCR analysis (Fig. 6a–c). Further, our results showed that treatment with IL-1β (10 ng/ml) resulted in phosphorylation of the p38 and p65-subunits of the NFκB complex (Fig. 6d), key proteins regulating expression of several inflammation associated genes such as MMPs, Cox-2 and several other pro-inflammatory cytokines. Of note, western blots further showed that treatment with NTG-101 suppressed IL-1β induced phosphorylation of both the p38 and p65-subunits of NFκB in rat IVD-NP cells within 30 min of treatment (Fig. 6d, Supplementary Fig. S4). Furthermore, with the reduction in inflammation in IVD-NP, treatment with NTG-101 induced NP-cell proliferation in vitro (> 50%) for up to 96 h as compared to no treatment control cells (NTC) cells (Fig. 6e). Treatment with NTG-101 resulted in phosphorylation of extra-cellular signal regulated kinase-1/2 (Erk-1/2, also known as p42/44, Thr202/Tyr204), Akt (Ser473 and Thr308) and receptor Smads including Smad-2 (Ser465/467) and Smad-3(423/425) proteins in rat IVD-NP cells in comparison to untreated controls (NTC, Fig. 6f, Supplementary Fig. S5).

## Discussion

Degenerative disc disease (DDD) significantly impacts the quality of life in patients suffering from this condition globally^45, 46^. However, only a few biological therapeutics have made their way from pre-clinical models into human clinical trials for treatment of DDD. Among these, an intra-discal injection of MSCs is currently in clinical trial studies (Phase 1/2/3) focusing on safety and efficacy of BMSCs and IVD-NP derived cells^35–38^. In an effort to better understand the mechanisms involved with cell-based therapy, we used a rat-tail disc injury model of DDD to compare the efficacy of mesenchymal stem cells (MSCs) derived from three different sources including rat cartilage (rCDSCs), bone marrow (rBMSCs) and human umbilical cord (hUCMSCs). We compared the results of stem cell transplants with the non-cellular therapeutic, NTG-101 containing a combination of rhCTGF and rhTGF-β1 within an excipient solution. The results of this study demonstrated differences in the regenerative effects of growth factor-based therapeutics (NTG-101) and MSC-based therapies in a pre-clinical rodent model of DDD. Injured rat tail IVD-NPs injected with NTG-101 showed the presence of notochordal and stem cells as well as increased expression of healthy ECM proteins (aggrecan, Col2A1). However, IVDs injected with rCDSCs showed limited regeneration potential with moderate expression of aggrecan and ColA1 but failed to restore notochordal and stem cells in injured IVDs. Additionally, the loss of NP cells and lower expression of aggrecan was observed in IVD-NPs injected with rBMSCs and hUCMSCs, in a manner akin to IVDs injected with vehicle. These findings clearly demonstrated the differences in the regenerative capabilities of MSCs depending on the source from which these cells are derived. These results also indicate the need to understand the complexities involved with transplantation of MSCs into a degenerative IVD that is typified by a pro-inflammatory, hypoxic, acidic, and reduced nutrient milieu.

Although rCDSCs, rBMSCs and hUCMSCs show mesenchymal characteristics, only IVDs injected with rCDSCs showed features comparable to a healthy, uninjured age-matched IVD-NPs. These CDSCs were derived from rat healthy articular cartilage from the knee, the milieu of which shares similarities with the IVD-NP niche (low nutrient supply, avascular and hypoxia). Since CDSCs have a similar origin and resemblance to chondrocyte-like cells (CLCs) within the IVD-NP, rCDSCs have a higher propensity to differentiate into NP-like cells and regenerate a healthy ECM in degenerate IVDs. In contrast, our study showed injured and degenerative IVDs injected with rBMSCs or hUCMSCs showed features of degenerative IVD-NPs such as reduced cellularity and an excess of fibrocartilaginous matrix. Among degenerative IVDs receiving an intra-discal injection of rBMSCs or human UCMSCs, 70% of the IVD-NPs showed a fibrocartilaginous, metaplastic IVD phenotype like the vehicle control. These results clearly demonstrated the differential ability of MSCs of variable origin to repair and regenerate a degenerating IVD. These considerations are particularly relevant given the oxygen rich, nutrient and growth factor enriched micro-environments from which rBMSCs or hUCMSCs are derived. In contrast, several studies have suggested that BMSC transplantation reverses IVD degeneration^33–38^. The characteristics of BMSCs including low immunogenicity, ability to differentiate into NP-like cells, and possible capacity to preserve the structure and function of IVD may characterize them as a preferred choice for transplantation. It has been reported that an intra-discal injection of BMSCs induced an increase in the endogenous cell population in vivo, suggesting BMSCs promoted cell survival and proliferation as well as prevented apoptosis in a degenerative IVD-NP^32–34^. By tracking the fate of GFP-tagged BMSCs, Yang et al.^47^ showed that a proportion of BMSCs could survive up to 24 weeks in the degenerated murine intervertebral discs and arrest the progressive degeneration of the IVD-NP through self-differentiation and stimulatory effect on endogenous NP-cells. However, the decreased number of BMSCs observed at 24 weeks post-injection suggested that the degenerative environment may be detrimental to the survival of the BMSCs in IVD-NP.

Several studies have investigated and proposed a bidirectional transfer of proteins between MSCs and NP cells that may provide a new mechanism for the interaction between MSCs and NP cells^39, 40, 48–52^. To understand the impact of the IVD-NP microenvironment, in vitro co-culture experiments have been used to study the possible influence of the respective cells upon each other. Cytokines and/or growth factors secreted from MSCs can stimulate and regulate the viability and function of NP cells in their proximity and vice versa. Granulocyte colony-stimulating factor (GM-CSF), macrophage colony-stimulating factor (M-CSF), leukemia inhibitory factor (LIF) and interleukins (IL-6, IL-10 and IL-11) are constitutively expressed by MSCs^52^. In addition, MSCs have also been reported to secrete insulin growth factor-1 (IGF-1), epithelial growth factor (EGF) and several members of transforming growth factor beta (TGF-β) superfamily including BMP-2, BMP-4, BMP-6, BMP-7, and TGF-β1^48–50^. Richardson et al.^51^ showed a sharp increase in the expression levels of Sox9, aggrecan, collagen I, collagen II, and collagen VI genes in BMSCs after a 7-day cell–cell contact with NP cells. In addition, direct cell–cell contact with autologous human BMSCs enhanced the cell proliferation and proteoglycan synthesis in IVD-NP cells^51^. These results suggested that paracrine signaling and interaction between MSCs and NP cells benefit both the biological activities of NP cells and the differentiation of MSCs.

**Figure 7:**
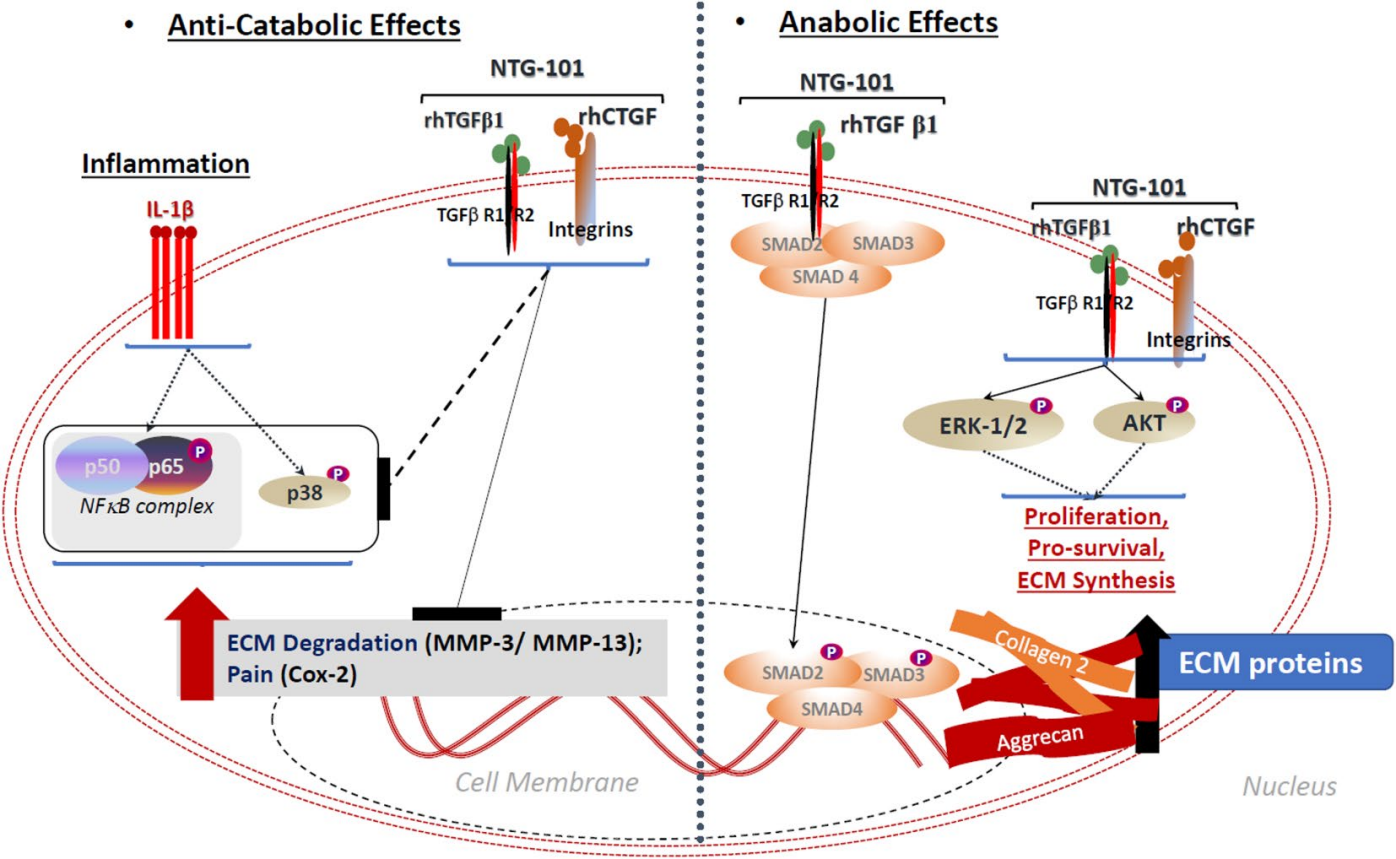
Model for mechanism of action (MoA) of NTG-101 in IVD-NP cells. Treatment with NTG-101 suppressed inflammation induced expression of matrix degrading enzymes (MMP-3. MMP-13), pain related enzyme, Cox-2 and expression of pro-inflammatory cytokines (IL-1β, IL-6 and IL-8) in rat, canine and human IVD NP cells in vitro and in vivo^27, 28^. Treatment with NTG-101 also suppressed phosphorylation of p38 and p65 subunit of NFκB complex, key regulators of inflammation in IVD-NP cells. Along with suppression of inflammation, NTG-101 also initiated ECM synthesis (Aggrecan, Col2A1), promoted cell survival and stemness in rat caudal IVD-NP post-injury. NTG-101 components, rhTGF-β1 and rhCTGF work synergistically under hypoxia resulting in regeneration of a degenerative disc. Combination of rhTGF-β1 and rhCTGF activates Smad-2/3 dependent signaling which regulate synthesis of GAG, PG and other important ECM molecules withing IVD-NP. Further, phosphorylation of Erk-1/2 (p42/44) and Akt in Smad-dependent or independent ways promote cell survival and proliferation in IVD-NP cells.

However, the engraftment and possible change in stem cell phenotype post-transplantation, as well as the integration of these transplanted cells within the IVD-NP remains controversial, with little evidence of cellular survival. Therefore, it raises the question that if the restorative effects of stem cells are dependent on secreted factors, would it not be preferable to deliver the requisite proteins more directly and avoid the risks of cellular transplant? The present study demonstrated that a single injection of NTG-101, containing a combination of rhCTGF and rhTGF-β1, provided superior regenerative potential in a pre-clinical model of DDD as compared to MSCs derived from three different sources (cartilage, bone marrow and umbilical cord). Our data showed that transplanted MSCs do not survive and integrate within the harsh, inflammatory microenvironment of the degenerative IVD. These results suggest that a minimally invasive intervention capable of inducing a regenerative effect upon the IVD without the associated risks of cell-based therapy may prove to be the most effective therapy for DDD. In our earlier reports, we showed that NTG-101 plays an important role in the suppression of inflammation and the restoration of ECM proteins both in vitro and in in vivo^27, 28^. Among the components of NTG-101, both rhCTGF and rhTGF-β1 are known for their anti-inflammatory and cell growth promoting functions. Our results demonstrated the anti-inflammatory and pro-anabolic effects of the combination of rhCTGF and rhTGF-β1 in rat, canine and human NP cells^27, 28^. Herein, we showed activation of multiple signaling cascades including suppression of IL-1β induced p38 and NFκB signaling and activation of pro-anabolic pathways such as Smad-2/3, Erk-1/2 and PI-3K/Akt leading to the development of an anabolic environment that catalyzes regeneration in IVD-NPs (Fig. 7). Transforming growth factor-β1 (TGF-β1), an important component of NTG-101 initiates multiple cellular signaling pathways by binding to and activating its specific cell surface receptors, TGF-β receptors-R2/R1, that possess an intrinsic serine/-threonine kinase activity^53–55^. These activated receptors stimulate the phosphorylation of receptor Smad proteins, Smad-2 and Smad-3 which form complexes with other co-Smads like Smad-4^53–55^. The hetero-trimeric complex of Smad-2/3/4 accumulates in the nucleus and regulate the transcription of several target genes involved in the development of a healthy extra-cellular matrix (aggrecan, Collagen 2A) and other proteoglycans^56, 57^. In addition, TGF-β1 is also known to induce cell proliferation and activate pro-survival signaling by activating non-Smad pathways involving phosphorylation of Erk-1/2 and Akt in a Smad-dependent or independent manner via TGF-β activated kinase 1 (TAK1) in cell type specific manner^58, 59^. Both the canonical and non-canonical TGF-β signaling pathways have been reported to promote glycosaminoglycans (GAG) and proteoglycan (PG) biosynthesis in NP cells of the disc^59^. Further, TGF-β growth factor signaling and tonicity-responsive enhancer binding protein (TonEBP), a transcription factor that regulates cellular osmolarity in NP cells work synergistically to maintain GAG and PG biosynthesis in NP cells^59^. In addition, rhCTGF also acts synergistically with rhTGF-β1 and hypoxia in regulating proteoglycan synthesis in IVD-NPs, supporting our in vivo results that treatment with the combination of these growth factor enhanced viability and ECM protein synthesis, while suppressing inflammation in vitro and in pre-clinical animal models of DDD^27, 28^. Our results strongly suggest that a single injection of NTG-101 into the degenerative disc can overcome the injurious and pro-inflammatory effects conferred by needle puncture injury and induce repair.

In conclusion, our results demonstrated that an intra-discal injection of NTG-101 into the degenerative IVD-NP conferred superior results as compared to an injection of MSCs (rCDSCs/rBMSCs/hUCMSCs). The NTG-101 injection promoted the maintenance of a healthy IVD-NP in terms of its morphology and cellular phenotype promoting the preservation of notochordal and stem cells in comparison to IVDs injected with MSCs alone that showed limited regenerative potential.

## Materials and methods

### Animals and ethics statement

All protocols were performed in accordance with policies and guidelines of the Canadian Council on Animal Care (CCAC) and approved by the Institutional Animal Care Committee of University Health Network (UHN), Toronto, Ontario, Canada. The study was carried out in compliance with the ARRIVE guidelines. Green fluorescent protein (GFP) expressing Wistar rats (strain: Wistar-Tg (CAG-GFP)184Ys) were sourced from The National BioResource Project for Rat in Japan (NBRP-Rat No: 0273). GFP-Wistar rats were housed and bred within the Animal Resource Center (ARC), University Health Network (UHN), Toronto, Ontario, Canada. In addition, wild type Wistar rats (12-weeks old) were obtained from Charles River Laboratories and housed at the Animal Resource Center (ARC), University Health Network (UHN), Toronto, Ontario, Canada for intradiscal injections as described below.

### Isolation and cell culture of rat mesenchymal stem cells (rMSCs)

Rat cartilage derived stem cells (rCDSCs) and bone marrow stem cells (rBMSCs) were derived from 12-week-old GFP-expressing Wistar rats (n = 8), following humane euthanasia using CO_2_ asphyxiation. Rat CDSCs were cultured in serum free media in low adherent cell culture flasks under hypoxia (3.5% O_2_) at 37 °C as suspension culture (Fig. 1a). While rBMSCs were cultured in adherent cell culture flasks in complete growth medium containing Advanced Dulbecco’s Modified Eagles Medium (AMDEM), fetal bovine serum (FBS, 10%), penicillin–streptomycin (1×) and glutamax (1×) under normoxia i.e., 5% CO_2_ at 37 °C (Fig. 1b). See Supplementary data for detailed protocol. These cells were characterized for MSC cell surface markers, pluripotency and lineage differentiation as described below.

### Cell culture of human umbilical cord derived stem cells (hUCMSCs)

Human Umbilical Cord Derived Stem cells (hUCMSCs) were purchased from American Type Cell Culture (ATCC, PCS­500­010). Cells were cultured in adherent flasks containing low serum MSC-growth media (ATCC PCS-500-030) and supplement (ATCC PCS-500-040) obtained from ATCC, under normoxia i.e., 5% CO_2_ at 37 °C as per manufacturer’s instructions. Human UCMSCs were characterized by ATCC for MSC markers and their differentiation into osteogenic, chondrogenic and adipogenic lineages^60^.

### Isolation, cell culture and treatment of rat caudal IVD-NP cells

Healthy rat caudal IVDs were removed aseptically from 32-week-old Wistar rats. The nucleus pulposus (NP) was separated and enzymatically digested according to our established methods^27, 28^. The next day, the cells were filtered with a 70 μm cell strainer and cultured within a hypoxic incubator (NuAire, MN, USA) in 3.5% O_2_, 5% CO_2_, 37 °C in Advanced Dulbecco’s modified Eagle’s medium (ADMEM) supplemented with 8% fetal bovine serum (FBS), penicillin and streptomycin (100 U/mL) until passage (P2) as described earlier^27, 28^. The NP cells obtained from rat IVDs were pooled together for treatment with respective agents as follows. The cells were either cultured in serum free DMEM used as no treatment controls (NTC) or treated with NTG-101, IL-1β (10 ng/mL) or IL-1β + NTG-101 for various time points under hypoxic conditions. The effect of NTG-101 on cell viability, expression of catabolic genes and associated cell signaling proteins regulating ECM synthesis or degradation, inflammation and pain in presence or absence of pro-inflammatory cytokine, IL-1β in NP cells was determined using qPCR and/or Western blotting as described below (also see “Supplementary Data S1” for details).

### RNA isolation, reverse transcription and quantitative real time PCR (qPCR)

Total RNA was isolated from spheroids of rCDSCs and adherent rBMSCs and hUCMSCs (passage, P2/3) using Trizol following manufacturer’s instructions. In addition, total RNA was also isolated from rat caudal IVD-NP cells treated with NTG-101, IL-1β (10 ng/ml), IL-1β (10 ng/ml) + NTG-101 and no treatment controls (NTC). See Supplementary data for detailed protocol. Total RNA was quantified using a NanoVue Plus (Biochrom, MA). cDNA was prepared from total RNA (~ 1 µg) using iScript Reverse Transcriptase (Biorad, CA). We determined the expression of mesenchymal stem cell surface markers (CD29, CD44, CD90, CD105, CD133, CD166), pluripotency markers (Oct4, Sox2), chondrogenic marker (Sox9), notochordal cell marker (Brachyury) and the hematopoietic progenitor cell markers (CD34, CD45) in MSCs using species and gene specific primers with the SYBR Green reagent (Thermo Fisher Scientific, ON) using quantitative real time-Polymerase Chain Reaction (qPCR) on the ABI 7900HT 96-well Fast block machine. For control and treated rat IVD-NP cells, expression of catabolic genes including MMP-3, MMP-13 and Cox-2 was also determined using qPCR. The sequence of the primers used is given in Supplementary data (Supplementary Table S1a,b). To check the specificity of the amplification products, melt curve analysis was carried out after each reaction. Species specific (rat/human) hypoxanthine phosphoribosyl-transferase (HPRT) gene expression was used as an internal control for normalization. No template controls, i.e., master mix without cDNA, was used as negative controls for the qPCR experiments.

### Immunofluorescence (IF)

20 × 10^3^ cells (rCDSCs/rBMSCs) were plated on Matrigel (BD Biosciences, CA) coated glass coverslips in 4-well plates containing ADMEM, fetal bovine serum (FBS, 8%) and penicillin/streptomycin (1X). After 24 h, media was discarded, cells were washed with PBS (1×, pH = 7.2) and fixed with paraformaldehyde (4%) for 30 min. Cells were washed with PBS (1×, pH = 7.2) after fixation, and incubated with goat serum (10%) for 1 h at room temperature. Immunocytochemical analysis was performed on cells using specific antibodies for mesenchymal cell surface markers (CD44, CD133, CD166), pluripotency markers (Sox2 and Oct4), notochordal cell marker (Brachyury), and chondrocyte marker (Sox9). Protein expression and localization was determined using Alexa 568 as fluorophore (Thermo Fisher Scientific, ON) and 4′,6-diamidino-2-phenylindole (DAPI) for nuclear staining. Images were captured using Confocal Laser Scan Microscopy (CLSM) using Olympus IX81 Inverted Microscope at Advanced Optical Microscopy Facility (AOMF), University Health Network (UHN), Toronto, Canada.

### Differentiation of rCDSCs and rBMSCs in osteogenic, chondrogenic and adipogenic phenotype

Rat CDSCs and BMSCs were plated in monolayers on glass coverslips in a 4-well plate in ADMEM containing FBS (8%) and penicillin–streptomycin (1×) for 24 h. Cell culture media was discarded, washed with PBS (1×, pH = 7.2, 3 times) and then treated with the appropriate differentiation medium following manufacturer’s instructions. For induced osteogenic differentiation in the cells using differentiation media containing osteogenesis supplement (Thermo Fisher Scientific, ON) for 21 days following manufacturer’s instructions and subsequently stained with Alizarin red (Millipore-Sigma, ON). For chondrogenic differentiation, cells were treated with chondrogenic media containing chondrogenesis supplement (Thermo Fisher Scientific, ON) for 2 weeks. To determine the differentiation of cells into chondrocytes, cells were stained with Safranin O staining (Millipore-Sigma, ON). For adipogenic differentiation, cells were cultured for 2 weeks with adipogenesis inducing medium containing adipogenesis supplement (Thermo Fisher, CA). Lipid droplets were revealed by staining with Oil Red O (Millipore-Sigma, ON).

### Intra-discal injection of vehicle, NTG-101 and MSCs (rCDSCs/rBMSCs/hUCMSCs) in pre-clinical rodent model of DDD

We used our established image-guided rat-tail needle puncture injury model of DDD using 12-week old female Wistar rats (n = 30), 5-caudal discs/animal using fluoroscopic image guidance as described earlier^27, 28^. We have previously shown that 10-week post needle puncture injury, the IVD assumes an ‘end-stage’ fibrocartilaginous degenerative phenotype^27^. In this study, we injured the rat-tail IVDs and waited 10 weeks before the PBS or therapeutic injections. For injuries, anesthesia was achieved using isofluorane (5 L/min plus 1 L/min O_2_) and maintained at 3 L/min. Once deeply anaesthetized, the animal was affixed on a stereotactic procedure apparatus (Model 900, Kopf Instruments, CA, USA) with nose cone inhalation. For disc injury, we used a 26-gauge (G) needle (Hamilton Company, USA) mounted on a Hamilton syringe. The needle was advanced completely through the selected tail IVD under fluoroscopic guidance. The animals were then removed from the stereotactic apparatus and allowed to recover in a warmed cage. Ten weeks post injury, animals were randomized, and the injured discs were injected with 8.0 µL of vehicle (i.e., phosphate buffer saline, PBS, 1×, pH = 7.2), NTG-101 or 150,000 cells (rCDSCs/rBMSCs/hUCMSCs) suspended in PBS (1×, pH = 7.2) using a 32G needle under fluoroscopic guidance. At the end of the experiment (i.e., 20 weeks post-injury), animals were humanely euthanized using CO_2_ asphyxiation and each vertebral lumbar/caudal motion segment was dissected aseptically. The IVDs were harvested and fixed in formalin for histological and immunohistochemical analysis. Age-matched (32-week-old) healthy IVDs were obtained from rat tails that served as uninjured, healthy controls.

### Histological analysis

For histology, rat caudal IVDs (healthy or treated with potential therapeutic agents) were removed, decalcified using CalEx (Fisher Scientific, ON) and paraffinized using histological cassettes as described earlier^27, 28^. The tissue block was trimmed with a microtome (Leica Biosystems, CA) until tissue was exposed, followed by cutting 5$\mu$ thick ribbons containing 10–12 paraffin-embedded tissue sections. Each section was collected on a single slide and checked for integrity of the tissue on the slide using bright field microscope. The tissue sections were discarded if integrity of the section was compromised i.e., section showed empty IVD-NP without any cells, IVD was too small showing lack of optimum depth of the block for cutting. Further to ensure quality of the tissue sections, at least three sections (1st, middle and the last) were used for Safranin O staining for assessment of histology and proteoglycan content, followed by immunohistochemical staining of proteins using specific antibodies on concurrent sections. Histological grading of the IVD-NPs (injury followed by treatment) was carried out based on morphology, cellularity and Safranin O staining intensity in these paraffin embedded, tissue sections representing the histology of respective IVDs from each treatment group as described earlier^27, 28^. See Supplementary Table S3 for histological scoring criteria in rat IVDs. The histological scoring was done by three observers (ME, AM and HG), and scores were recorded independently. In the case of inter-observer variability of scores, all three observers reviewed scores and arrived at a final consensus score.

### Immunohistochemistry (IHC)

Immunohistochemical analysis was performed in serial sections for ECM proteins (Aggrecan, Collagen 2), the notochordal cell marker (Brachyury), stem cell marker (Oct4), chondrocyte marker (Sox 9) and Green Fluorescent Protein (GFP) using the Vectastain^tm^ rabbit or mouse kit (Vector Labs, ON). Briefly, after Safranin-O staining, subsequent serial tissue sections were deparaffinized in xylene followed by hydration in gradient alcohol. Antigen retrieval was performed using microwave-based heat retrieval method. Thereafter, slides were washed three times with Tris-buffered saline (TBS, 1X, pH = 7.4) containing 0.025% Triton-X-100 (TBS-T), followed by blocking with appropriate serum, provided in the kit. The slides were then incubated with rabbit polyclonal or mouse monoclonal primary antibodies at appropriate dilutions for 1 h at room temperature followed by washing with TBS-T, three times). The sections were incubated with hydrogen peroxide (0.3% v/v) for 15 min to block endogenous peroxidase activity, followed by three washes with TBS-T. Tissue sections were incubated with the goat anti-rabbit or mouse secondary antibody at appropriate dilution for 30 min. Protein expression was detected using diaminobenzidine (DAB) as chromogen. The sections were counterstained with Meyer's hematoxylin and mounted with DPX mountant. For scoring, ECM and cytoplasmic staining for Aggrecan, Col2A1 and nuclear expression of Brachyury, Oct4 and Sox-9 was considered as positive staining for IHC analysis (Supplementary Table S4). The bright field sections were evaluated semi-quantitatively for % positivity and staining intensity in IVD-NP tissue by light microscopic examination using a Nikon bright field microscope (Nikon Eclipse TE2000-U).

### Cell viability assay

Rat IVD-NP (4000 cells per well) were plated in 96-well flat bottom plates to evaluate the effect of treatment with NTG-101 on cell viability in time dependent manner. Rat NP cells (passage, P2) were treated with NTG-101 in serum free media for 48–96 h and cell viability was determined using 3-(4,5-dimethylthiazol-2-yl)-2,5-diphenyltetrazolium bromide (MTT, Sigma-Aldrich, USA) as described earlier^27, 28^.

### Western blotting

For Western blotting, protein estimation was performed using Bradford assay (BioRad, CA). Briefly, equal amounts of rat IVD-NP cell lysates prepared using NP-40 lysis buffer containing protease and phosphatase inhibitors (Millipore-Sigma, ON), were subjected to Western blotting as described earlier^27^. Whole cell lysates (30 μg) were resolved on 10% or 4–15% gradient sodium dodecyl sulphate–polyacrylamide gels (SDS-PAGE, Biorad, CA) under reducing conditions and proteins were electro-transferred onto polyvinyledendifluoride (PVDF) membranes using Trans-Blot Turbo System (BioRad, CA). After blocking with 5% non-fat powdered milk (for non-phospho-proteins) or 1% bovine serum albumin (BSA, for phosphorylated signaling proteins) in Tris-buffered saline (TBS, 0.1 M, pH = 7.4), blots were incubated with rabbit polyclonal or mouse monoclonal primary antibodies at 4 °C overnight. Membranes were washed three times with Tween (0.1%)-Tris-buffer saline (TTBS) and then incubated for 2 h at room temperature with the respective horse radish peroxidases (HRP)-conjugated anti-IgG secondary antibodies (BioRad, CA), diluted as per the manufacturer’s instructions in 2% non-fat milk in TBS (pH = 7.2, 1×). Blots were washed three times with TTBS for 15 min, protein bands were detected by the enhanced chemiluminescence reagents (BioRad, CA) and images captured using GE AI600RGB Imager (Cytiva, MA). β-actin was used as a loading control for each experiment.

### Statistical analysis

For qPCR analysis, gene expression was evaluated based on Ct-values. Each sample was run in duplicate, and the fold change was calculated using 2^−(ddCt)^ method. For MSCs, histograms represent fold change of gene expression in rCDSCs and hUCMSCs with respect to rBMSCs. For viability assays and expression of catabolic genes (MMP-3, MMP-13 and Cox-2), histograms represent viability and expression in treated cells with respect to no treatment control (NTC) cells. For histological analysis, histograms showing average scores for morphology (M), cellularity (C), Safranin O staining intensity (I) and total score (M + C + I) were plotted for all variables considered in this study. For immunohistochemical analysis, total score was evaluated as the sum of score on % positivity and intensity in each tissue section as described earlier^27, 28^. All data are expressed as average score ± standard deviation (S.D.). Statistical analysis was performed using MS-Excel using 2-tailed Student’s t-test. P-value ≤ 0.05 was defined as statistically significant for all tests.

## Supplementary Information

Supplementary Information.
