# Supplementary material for: A comparative study of mesenchymal stem cell transplantation and NTG-101 molecular therapy to treat degenerative disc disease
Source: Sci Rep. 2021 Jul 20;11:14804. doi: 10.1038/s41598-021-94173-w (PMC8292352; doi:10.1038/s41598-021-94173-w)

Title:

A Comparative Study of Mesenchymal Stem Cell Transplantation and NTG-101 Molecular Therapy to Treat Degenerative Disc Disease

Authors: Ajay Matta[1], Muhammad Zia Karim[1], Hoda Gerami[1], Bettina Benigno[1], W. Mark Erwin[1,2,3]

Affiliations: [1]Notogen Inc., Toronto, Ontario, Canada.

[2]Dept of Surgery, University of Toronto, Toronto, Ontario, Canada.

[3]Canadian Memorial Chiropractic College, Toronto, Ontario, Canada.

Corresponding Author: *W. Mark Erwin DC, PhD

Email: mark.erwin@utoronto.ca; Tel: 1-647-243-5278

Short Title: NTG-101 vs. stem cell therapy for DDD

Key words: Degenerative Disc Disease, NTG-101, Mesenchymal Stem cells, Nucleus pulposus, Inflammation, Pre-clinical models, TGF-β1

SUPPLEMENTARY DATA

MATERIALS AND METHODS

Reagents and Antibodies. Recombinant human (rh) Connective Tissue Growth Factor (rhCTGF) and Transforming Growth Factor beta 1 (TGF-β1) were purchased from Peprotech (NJ, USA). Cell culture media Advanced Dulbecco's Eagle media (ADMEM). Neurobasal A media, fetal bovine serum (FBS), penicillin-streptomycin, B-27 supplement, Epithelial Growth Factor (EGF), Fibroblast Growth Factor-2 (FGF-2), heparin, insulin-transferrin, Trizol, were purchased from Thermo-Fisher (MA, USA). Putrescine, selenium and progesterone were purchased from Millipore (ON, Canada). Collagenase type II was purchased from Roche Biosciences (QC, Canada). Human Umbilical Cord Derived Stem cells (hUCMSCs), growth media and supplements were purchased from American Type Cell Culture (ATCC, VA, USA). Rabbit polyclonal antibodies for CD44, CD133, Sox2, Oct4, Brachyury, and rabbit monoclonal antibody for CD166, Sox9 was purchased from Abcam Inc. (ON, Canada). Mouse monoclonal antibody for detecting GFP-expression was purchased from Takara Biotech (CA, USA). For cell signaling proteins, rabbit polyclonal or monoclonal antibodies present in Cell Signaling type specific Antibody Sample Kits were purchased from Cell Signaling Technologies (New England Biolabs, CA). Stem cell differentiation kits, Alexa 568 fluorophore labeled goat anti - rabbit / anti - mouse antibodies were purchased from were also purchased from Thermo-Fisher (MA, USA). Vectastain ABC kits (rabbit / mouse) and chromogen, diaminobenzidine (DAB) for immunohistochemistry was purchased from Vector laboratories (ON, Canada).

Preparation of NTG-101. NTG-101 is a proprietary formulation containing a combination of rhCTGF (100 ng/ml) and rhTGF-β1 (10 ng/ml) proteins suspended in an excipient solution (ES). Stock solution of rhCTGF (100 µg/ml) was prepared in sterile water while rhTGF-β1 (10 µg/ml)

was dissolved in 0.1 M Citric acid. Both rhCTGF and rhTGF-β1 proteins were added in appropriate dilutions in excipient solution as described before [28].

Isolation and Cell Culture of Rat Cartilage Derived Stem Cells (rCDSCs). Following humane euthanization, 12 weeks old GFP - Wistar rats (n = 8), underwent hind limb dissection, and cartilage tissue was scraped from femoral head, femoral condyles and tibial plateau. Cartilage tissue was collected in 50 ml tubes in Advanced Dulbecco's Minimal Essential Media (ADMEM) containing fetal bovine serum (FBS, 4%), penicillin / streptomycin (1X, Thermo Fisher, MA USA), Collagenase type II (1 mg/mL, Roche Biosciences, QC, Canada) and incubated on a shaker at 37^0C for 18 - 20 hours. The next day, the cells were strained through a 70 µm filter (Millipore) and centrifuged at 800 xg for 5 minutes. The media was discarded, and the cell pellet was washed with 10 mL of phosphate buffer saline (PBS, 1X, pH = 7.2). Cells were counted using EVE automatic cell counter (NanoEnTek, CA) and plated as suspension culture in low adherent T75-cell culture flasks containing Neurobasal media (Thermo-Fisher, MA, USA), B-27 supplement (1X), 0.02% Epithelial Growth Factor (EGF), 0.02% Fibroblast Growth Factor-2 (FGF-2), 0.007% heparin, insulin-transferrin (1X), putrescine (0.01%), selenium (0.01%) and progesterone (0.01%, Millipore Sigma) under hypoxia (3.5% O2) at 37^0C. Cells were allowed to form spheroids / cell spheres in suspension cultures, mechanically dissociated in 1 mL Trypsin and re-cultured under clonal density conditions. Resulting spheres forming from single cells were then recovered and tested for stemness characteristics using gene specific primers and antibodies for MSC surface markers as described below.

Isolation and Cell Culture of Rat Bone Marrow Stem Cells (rBMSCs). Following euthanasia, 12-week-old GFP-Wistar rats (n = 8), we aseptically removed the both femurs, tibiae and the cancellous bone was reamed with a 21G needle into a sterile petri dish and washed 3 - 5 times with

sterile PBS (1X). Thereafter, the recovered bone marrow was flushed using 10 ml syringe onto a 70 µm filter mesh placed over a 50 ml tube containing 20 - 25 ml sterile PBS. The tube was then centrifuged at 500 xg for 5 minutes and the supernatant discarded. We then added 1 ml of ACK lysis buffer (Qiagen, CA, USA) and incubated for 10 minutes at room temperature. The tube was again centrifuged at 500 xg for 5 minutes and the supernatant was discarded. The cell pellet was resuspended in complete growth medium containing Advanced Dulbecco's Modified Eagles Medium (AMDEM), fetal bovine serum (FBS, 10%), penicillin-streptomycin (1X) and glutamax (1X). Cells were counted using EVE automatic cell counter (NanoEnTek, CA) and plated 50,000 cells / ml in T75 cell culture flasks. Cells were then cultured in incubator under normoxia i.e. 5% CO_2 at 37^0C (NuAire, MN, USA).

RNA Isolation. Cell spheres from rCDSCs were collected by centrifugation at 1000 rpm, 4^0C and media was discarded. The cell pellet was suspended in 1 ml of Trizol and mixed well by vortexing. For adherent cell cultures, rBMSCs and hUCMSCs, the media was discarded and 1 ml of Trizol was added in T25 cell culture flasks. Trizol was transferred into fresh RNAse free, eppendorf tubes and 200 µl of absolute ethanol was added. Tubes were inverted, mixed, and kept at room temperature for 5 minutes. The clear, aqueous phase was transferred from the top layer into a fresh eppendorf tube containing 500 µl of iso-propyl alcohol, mixed well and incubated for 10 minutes, followed by centrifugation for 30 minutes at 13000 rpm in a bench top centrifuge, at room temperature (RT). The pellet was washed with 1 ml of RNAse free 70% ethanol, dried and dissolved in RNAse free water. DNAse treatment was given using on column DNAse 1 provided in the kit (Qiagen, CA) following manufacturer's instructions.

Supplementary Figure Legends

Supplementary Figure S1. Stem cell differentiation into specific lineage. rCDSCs and rBMSCs were differentiated into osteogenic, chondrogenic and adipogenic lineages following standard differentiation protocols. Panel shows differentiation of rat (**a**) rCDSCs and (**b**) rBMSCs into osteogenic (Alizarin red stain), chondrogenic (Safranin O stain) and adipogenic lineages (Oil red O stain), Scale bar: 500 µm.

Supplementary Figure S2. Panel (**a**) shows expression of GFP in tissue sections of (**a**) GFP-rat tail IVD-NP used as a positive control. Panels (**b**) and (**c**) show no detectable expression of GFP or GFP-expressing rCDSCs / rBMSCs injected in rat tail, injured IVDs 20 weeks post injury (Scale bar: 50 µm).

Supplementary Figure S3. Immunohistochemical analysis of cell specific markers in in vivo rat tail needle puncture model of DDD. Panels show expression of (**a**) Col2A1, (**b**) nuclear Brachyury and (**c**) Sox9 in uninjured, healthy IVD-NPs and IVDs injected with vehicle, NTG-101 or MSCs (rCDSC, rBMSCs or hUCMSCs). All images were acquired using bright field Nikon microscope (Nikon Eclipse TE2000-U, Scale bar: 100 µm).

Supplementary Figure S4. Figure represents full -length Western blots for pp38, p38, pp65, p65 and β-actin in whole cell lysates prepared from rat IVD-NP cell lysates treated with IL-1β, IL-1β+NTG-101 or left untreated that served as no treatment controls (NTC).

Supplementary Figure S5. Figure represents full -length Western blots for pp42/44, total p42/44, pAkt (Ser), pAkt (Thr), pan-Akt, pSmad-2, total Smad-2, pSamd-3, total Smad-3 and β-actin used as loading controls in Western blotting using whole cell lysates prepared from rat IVD-NP cell lysates treated with NTG-101 or left untreated that served as no treatment controls (NTC).

Supplementary Table S1a. Rat gene specific primer sequences for qPCR

Gene	Forward	Reverse
**CD34**	GAACCGCCGCAGTTGGAGTC	CGTTCTCCTGAGCCCCTCGG
**CD45**	AAGGAAACTTGCTCCCCATCCG	TGAGGTTGGCACCATCGTCGG
**CD29**	CTGCACCGTTGGGGTTGGAGT	CTGAACACTGGGCCTTTGGGGA
**CD44**	CCCTGGCCACCAGTGATGGAG	TGAGGTCTCCTCGCAGGACCA
**CD90**	TGCAAGCTAGGGGAGCCCAG	GACGGCAGTCCAGTCGAAGGT
**CD105**	TCCGCTGCCGTCCCAAAGAG	GGTTCGCACGGGGATCCGAA
**CD133**	GGTTCCAAGACCCAGGCGGG	TGGCCTGATCCCCCAGGCAT
**CD166**	ACAGAAGCCTAAGAGAGGAGTTGT	TCCCAGGACAGCTTAGTAGGATGA
**Sox2**	CGCGGCGGAAAACCAAGACG	GCCGTTCATGTGCGCGTAGC
**Oct4**	AGGCCCGGAAGAGAAAGCGG	CCCTTCTGGCGCCGGTTACA
**Sox9**	ACACGTTCCCCAAGGGCGAG	TCATGGGCCGCTTGACGTGT
**Brachyury**	GCAGGCTCTTGGGACAAGGGG	AGCCTCGTCTCTGTTCCTGGC
**MMP-3**	TTTGGCCGTCTCTTCCATCC	GCATCGATCTTCTGGACGGT
**MMP-13**	TGCATACGAGCATCCATCCC	CTCAAAGTGAACCGCAGCAC
**Cox-2**	ATCAGAACCGCATTGCCTCT	GCCAGCAATCTGTCTGGTGA
**HPRT**	ACCAGTCAACGGGGGACATA	TTGGGGCTGTACTGCTTGAC

Supplementary Table S1b. Human gene specific primer sequences for qPCR

Gene	Forward	Reverse
**CD34**	AGAAAGGCTGGGCGAAGACCC	CCCTCGGTTCACACTGGCCT
**CD45**	ACCAGGAATGGATGTCGCTAATCA	GGGGCCTGTAAAAGTGTCCTGC
**CD29**	GACGCCGCGCGGAAAAGATG	GCACCACCCACAATTTGGCCC
**CD44**	CAGGTTCCCCGACCCACGTC	GCGAGCGAAGGACACACCCA
**CD90**	GCAGGCGGCCATGGGTTAAAG	CTGGGCTGGCACCTTCTGGTC
**CD105**	CCCTGCTGCCGGTCATACCA	TGCTGTCCGAAGGATGGGCG
**CD133**	GCATCCCTCGCCTCAAGCCA	GGTGCCTCCTGCCTCAGAGC
**CD166**	TCCAGAACACGATGAGGCAGACG	ACGACACCAGCAACAAGGGCA
**Sox2**	GGCCGTTCATCGACGAGGCT	GCTCGCCATGCTATTGCCGC
**Oct4**	AGTAGTCCCTTCGCAAGCCCT	GGGCGAGAAGGCGAAATCCGA
**Sox9**	GCCGACTCGCCACACTCCTC	CGCGGAAGTCGATAGGGGGC
**Brachyury**	TTGACTGCTCTGCCCCCTAGA	TCACAAAAGGAGGGGCTTCACT
**HPRT**	CGAGATGTGATGAAGGAGATGGG	ATCCAGCAGGTCAGCAAAGAAT

Supplementary Table S2. Gene expression analysis in rat (rBMSCs / rCDSCs) and human (hUCMSCs) using species specific primers for qPCR

Gene Markers	Cell Type / Gene Expression	rBMSCs	rCDSCs	hUCMSCs
Mesenchymal Stem Cell Markers	CD29	+	+	+
	CD44	+	+	+
	CD90	+	+	+
	CD105	+	+	+
	CD133	+	+	+
	CD166	+	-	+
Hematopoietic Markers	CD34	-	-	-
	CD45	-	-	-
Pluripotency Markers	Sox2	+	+	+
	Oct4	+	+	+
Chondrocyte Marker	Sox9	+	+	+
Notochordal Cell Marker	Brachyury	-	-	-

Supplementary Table S3a. Scoring criteria for rodent IVD-NP histology using Safranin O stained tissue sections.

Score	Morphology	Cellularity
**1**	Round, comprising at least half of the disc area	>80% cellularity i.e. rich in notochordal cells.
**2**	Round or irregularly shaped comprising one quarter to half of the disc area	50-80% cellularity with ECM
**3**	Irregularly shaped comprising less than one quarter of the disc area.	10-50% cellularity with cell clusters separated by dense areas of proteoglycan rich ECM.
**4**	NP absent / very small due to hyper-proliferative AF.	Fibrocartilaginous (FC)-NP with <10% cellularity

Supplementary Table 3b. Scoring criteria for Safranin O staining intensity

Score	% Intensity
**1**	Mild / Faint
**2**	Moderate
**3**	Strong

Supplementary Table S4. Scoring criteria for immunohistochemistry (IHC)

Score	% Positivity	% Intensity
**0**	None	None
**1**	1 - 10%	Mild / Faint
**2**	10 - 50%	Moderate
**3**	>50%	Strong

Supplementary Figure S1.

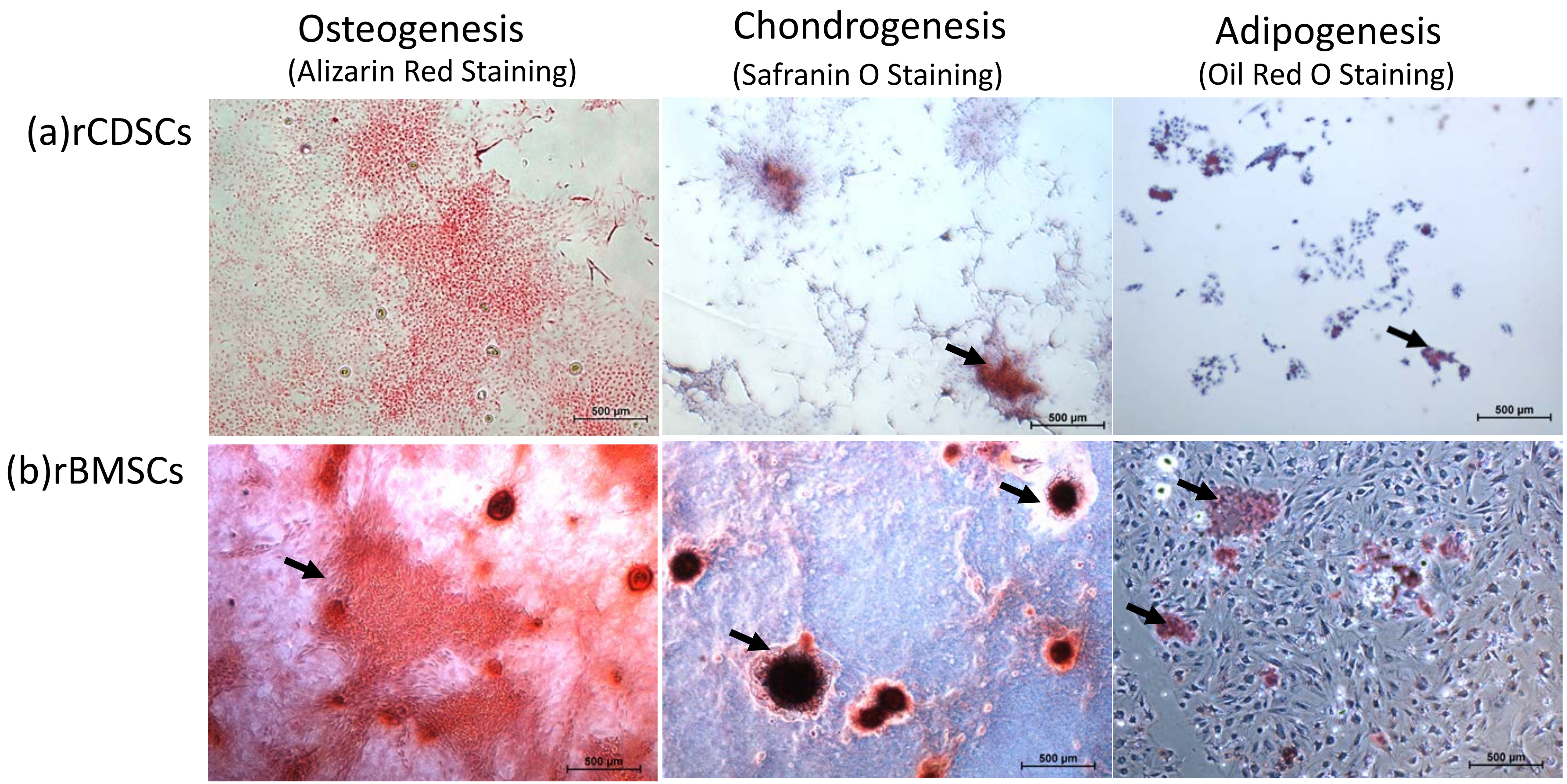

Supplementary Figure S2.

(a) GFP-Rat Caudal IVD-NP (b) Rat Caudal IVD-NP (rCDSCs) (c) Rat Caudal IVD-NP (rBMSCs)

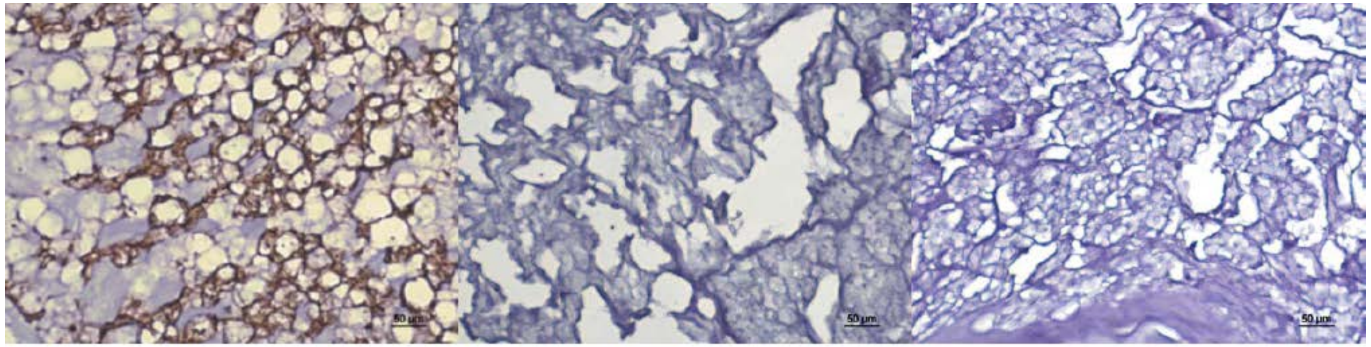

Supplementary Figure S3.

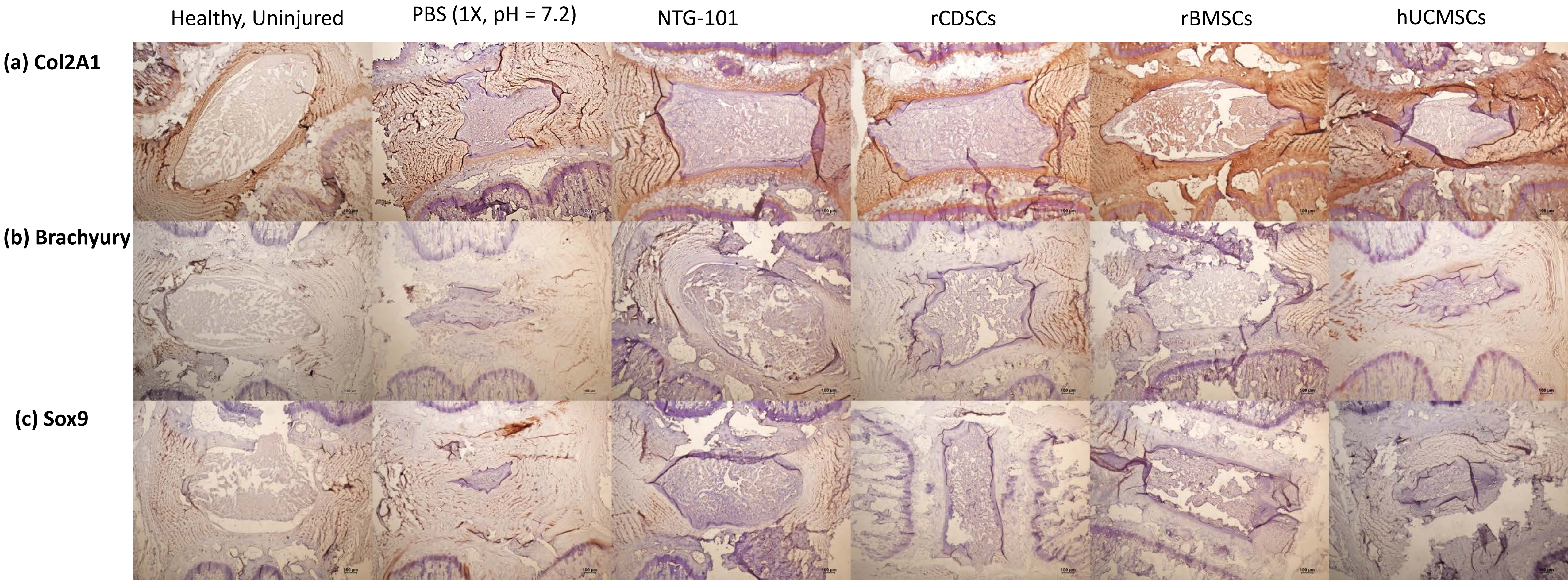

Supplementary Figure S4.

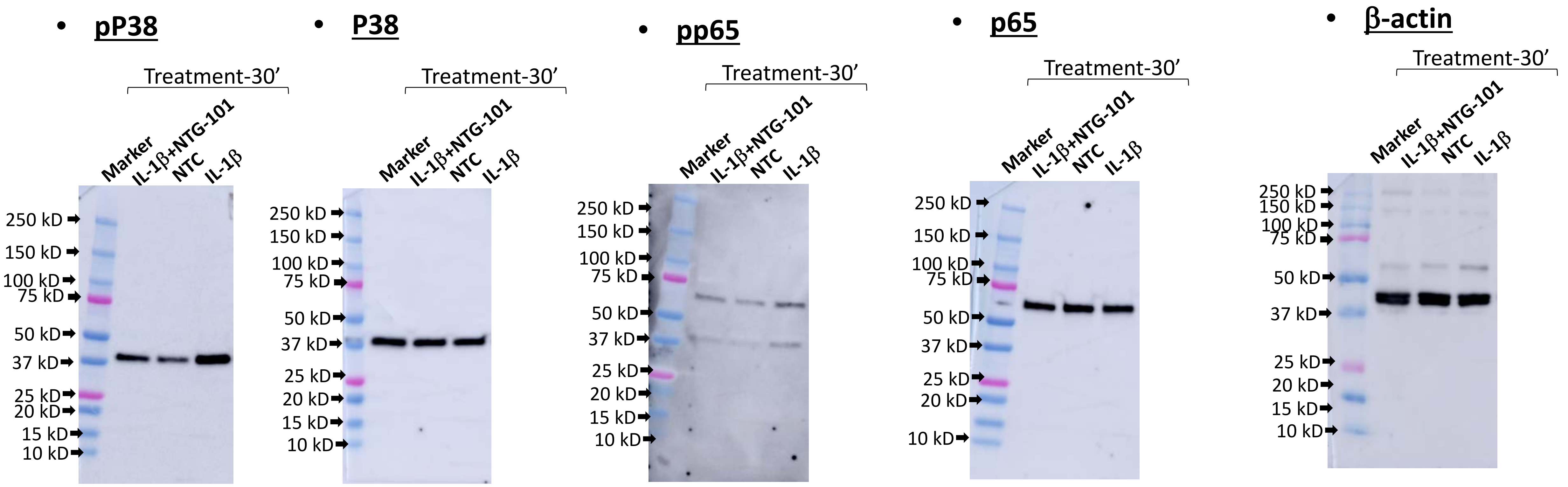

Supplementary Figure S5.

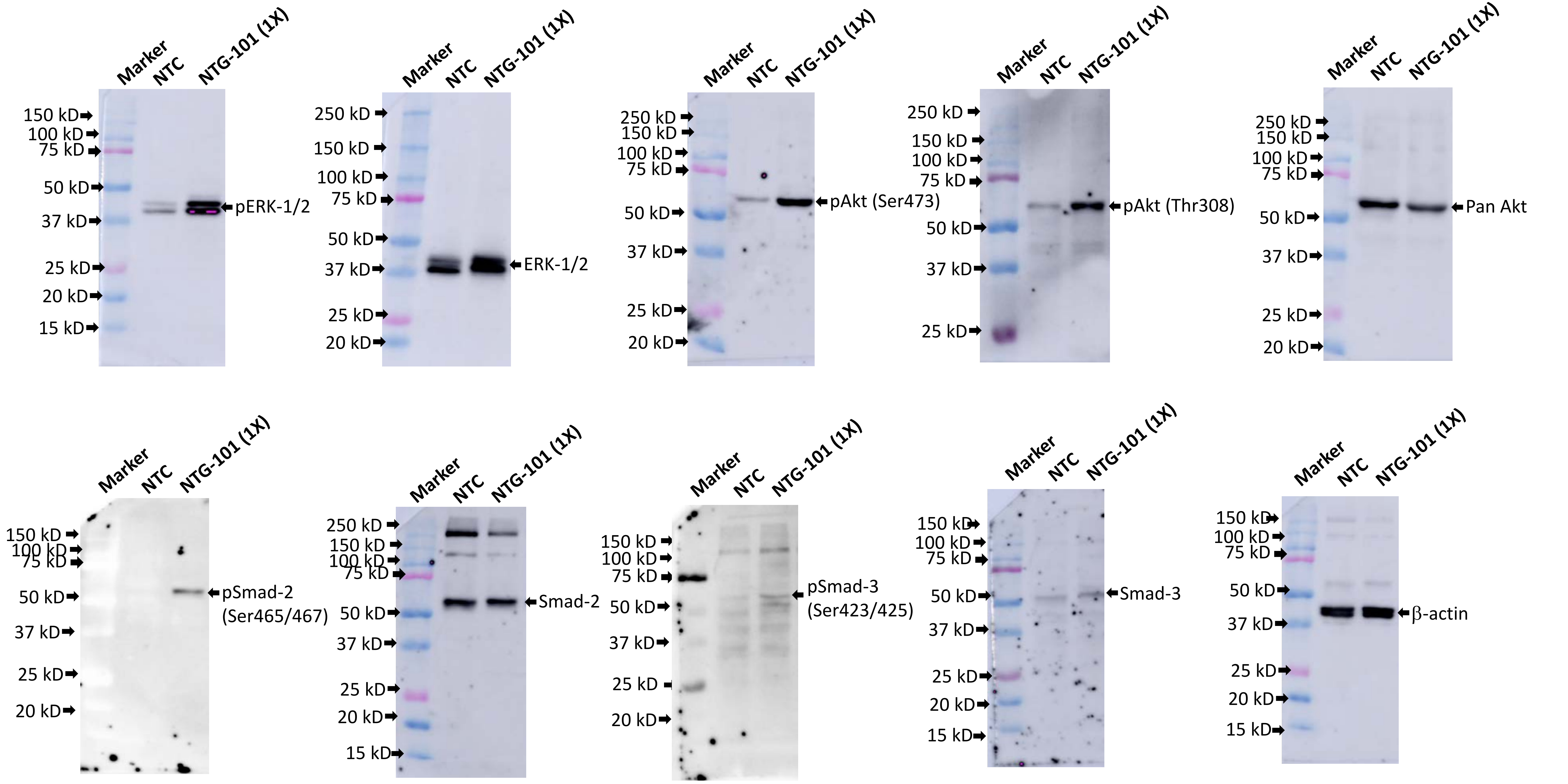

Supplement: Supplementary file 1 — Supplementary Information. [file 41598_2021_94173_MOESM1_ESM.pdf]
